# Combined Forehead and Temporal Lifting: An Innovative Approach to Lanluma V Treatment

**DOI:** 10.1111/jocd.16583

**Published:** 2024-09-25

**Authors:** Larry Wu, Giovanni Salti

**Affiliations:** ^1^ iCare Medical Centre Singapore; ^2^ Istituto Medlight Firenze Italy

**Keywords:** forehead lift, Global Aesthetic Improvement Scale, Lanluma V, non‐scarring alopecia, nonsurgical facial rejuvenation, poly‐l‐lactic acid, temporal lift

## Abstract

**Background:**

Excess skin laxity over the upper face can contribute to aging over the mid and lower face. We describe an innovative nonsurgical technique of facial rejuvenation by injecting Lanluma V over the scalp's vertex and parietal regions. Lanluma V is a poly‐l‐lactic acid (PLLA)‐based collagen stimulator which contains 210 mg of PLLA, distributed by Sinclair Pharmaceutical. Lanluma V works by stimulating collagen regeneration to provide support for the treated area.

**Method:**

A retrospective review of 12 consecutive patients treated with Lanluma V over the vertex and parietal regions of the scalp to achieve nonsurgical rejuvenation of the upper, middle, and lower thirds of the face was conducted. The patients were treated over two sessions, 1 month apart. The treated patients were reviewed by a plastic surgeon and rated under the Global Aesthetic Improvement Scale (GAIS) 6 months after treatment.

**Results:**

The patients achieved an overall average of 1.16 grade improvement in GAIS. The average follow‐up period is 6 months following completion of treatment. There was no reported incidence of non‐scarring alopecia, which has been reported in the use of other, more viscous fillers such as calcium hydroxyapatite or high G' hyaluronic acid.

**Conclusion:**

This innovative method of combined forehead and temporal lifting with Lanluma V allows for an average 1.16 grade improvement in GAIS. There is no reported incidence of non‐scarring alopecia, which has been associated with other fillers.

## 1 Introduction

The pursuit of facial rejuvenation through nonsurgical methods has become increasingly popular in recent years. Facial aging is a complex three‐dimensional process involving changes in the skin, soft tissue, and underlying bone layer [1]. The aging of the skin can be affected by both intrinsic and extrinsic factors leading to changes in skin texture, tone, and quality. Intrinsic aging is a natural consequence of chronological aging and is primarily determined by genetic factors [2]. Extrinsic factors include ultraviolet (UV) radiation, smog, stress, and lifestyle factors such as smoking and diet can accelerate aging [3]. The impact of aging on our skin can accelerate the formation of wrinkles and the loss of skin elasticity. One of the fundamental causes of aging is the loss of collagen due to decreased synthesis by dermal fibroblasts, decline in number of dermal fibroblasts and also increased breakdown by matrix metalloproteinases [4]. The PLLA in Lanluma V is an ideal agent for addressing collagen depletion by stimulating the synthesis of collagen by fibroblasts. Our innovative technique of stimulating collagen production in the scalp over the vertex and parietal regions provides structural support and reduces the effect of aging over the upper, middle, and lower face.

## 2 Method

### 2.1 Assessment

We conducted a retrospective review of 12 patients from 2022 to 2023 who had received the combined forehead and temporal lift over two sessions using Lanluma V, and assessed them using GAIS. The assessor is a plastic surgeon who is not involved in performing the treatment. The patients were initially assessed for suitability for the technique of forehead and temporal lifting as a method of facial enhancement and rejuvenation. They were informed of the need for two treatments, 1 month apart. In addition, they were informed of the occurrence of post‐procedural swelling due to the need for injection of tumescent solution. The patients were encouraged to perform self‐massage over the treated area every morning and night, for 10 min each time, for 10 days. Pre‐ and post‐procedural photos were taken, and the patients underwent follow‐ups 1 month after each treatment.

### 2.2 Technique

Pre‐procedural markings are performed with surgical marker, and local anesthesia consisting of 2% lignocaine as well as 1:200 000 adrenaline is injected to anesthetize the entry points. Lanluma V is reconstituted with 15 mL of sterile water for dilution (SWFI), shaken for 15 min, and allowed to stand for 1 h prior to treatment, and 1 mL of lignocaine is added to the cocktail before treatment for analgesia.

### 2.3 Scalp Vertex Treatment

**FIGURE 1:**
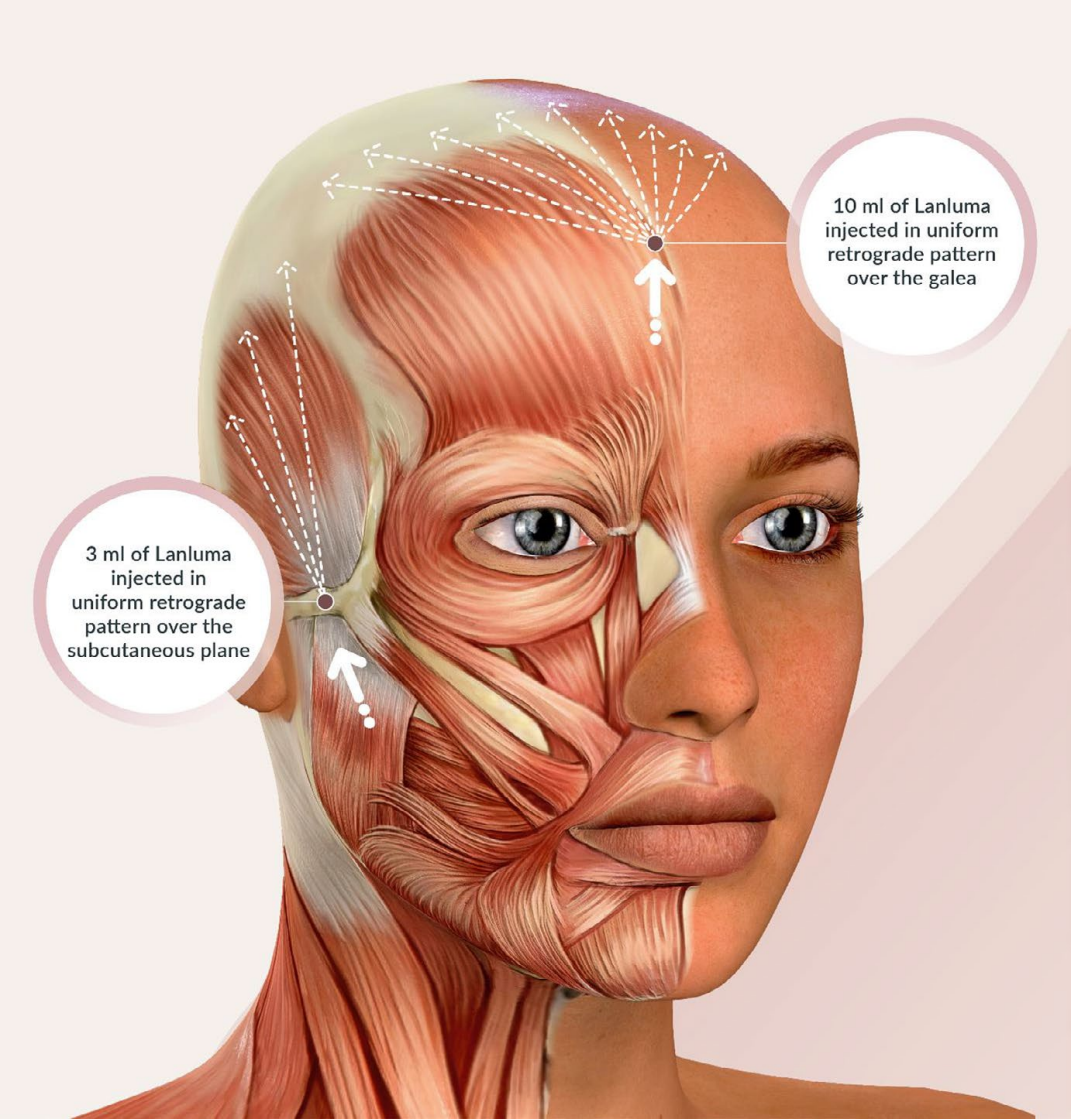
Black dots showing the entry points for treatment over the vertex and the parietal region. White arrows showing the direction of forehead and temporal lifting following regeneration of collagen.

Tumescent solution consisting of 10 mL of normal saline with 1.1 mL of 2% lignocaine and 1:200 000 adrenaline is infused at the supraperiosteal level to create an envelope for treatment with Lanluma V. Ten milliliter of reconstituted Lanluma V is injected over the vertex in a fanning retrograde manner over the galea aponeurotica layer using a 22‐G 70 mm cannula (See Figure 1).

### 2.4 Parietal Scalp Treatment

The entry point for the parietal treatment is over the zygomaticotemporal suture, and 3 mL of Lanluma V is injected in the deep subcutaneous plane over the parietal region in a linear fan shaped manner. Care is taken to avoid injuring the superficial temporal artery. The Lanluma V is injected using a 22G 70 mm cannula in a retrograde fashion. Patients are reminded to massage the area twice a day, 10 min each time, for 10 days. The patient is informed that the immediate swelling is an effect of injected Lanluma V and the optimal result will be observed in 4–6 weeks after treatment.

### 2.5 Assessment

The photo of patients are then assessed for aesthetic results 6 months following treatment and scored by a plastic surgeon for aesthetic improvement.

## 3 Results

The patients scored, on average, a 1.16 grade improvement in their GAIS scores (See Table 1), as assessed by a plastic surgeon. All patients experienced swelling of the forehead following treatment and this can be attributed to the pretreatment with 10 mL of tumescent solution. The post‐procedural swelling resolved within 1 week with conservative management. There was no incidence of non‐scarring alopecia in the parietal region that has been described with the use of hyaluronic acid or calcium hydroxyapatite [5].

**TABLE 1:** Clinical characteristics of the patients, Global Aesthetic Improvement Scale (GAIS) evaluation, and grade improvement.

Patient	Age	Global Aesthetic Improvement Scale	Improvement
A	47	3	1
B	76	4	0
C	49	3	1
D	47	3	1
E	42	2	2
F	35	2	2
G	61	4	0
H	45	3	1
I	48	4	0
J	66	2	2
K	32	2	2
L	49	2	2
		Average improvement	1.167

## 4 Discussion

In the dynamic world of nonsurgical aesthetic enhancements, advancements in techniques and materials continually reshape the landscape of aesthetic procedures. Our article demonstrates a novel technique incorporating the effects of scalp vertex volumization to complement the effects of temporal lifting. The treatment over the scalp vertex is preceded by infusion of tumescent solution over the galea to hydrodissect an envelope prior to injection of Lanluma V biostimulator. This allows for greater patient comfort and a more even distribution of Lanluma V to avoid the formation of nodules. This technique of injecting over the vertex provides support to address skin laxity over the forehead. Care is taken to avoid injecting into the forehead convexity, although PLLA has been proposed in an Asian consensus paper as a treatment modality for forehead convexity [6]. Forehead injection of PLLA is not performed due to the risk of persistent nodules.

**FIGURE 2:**
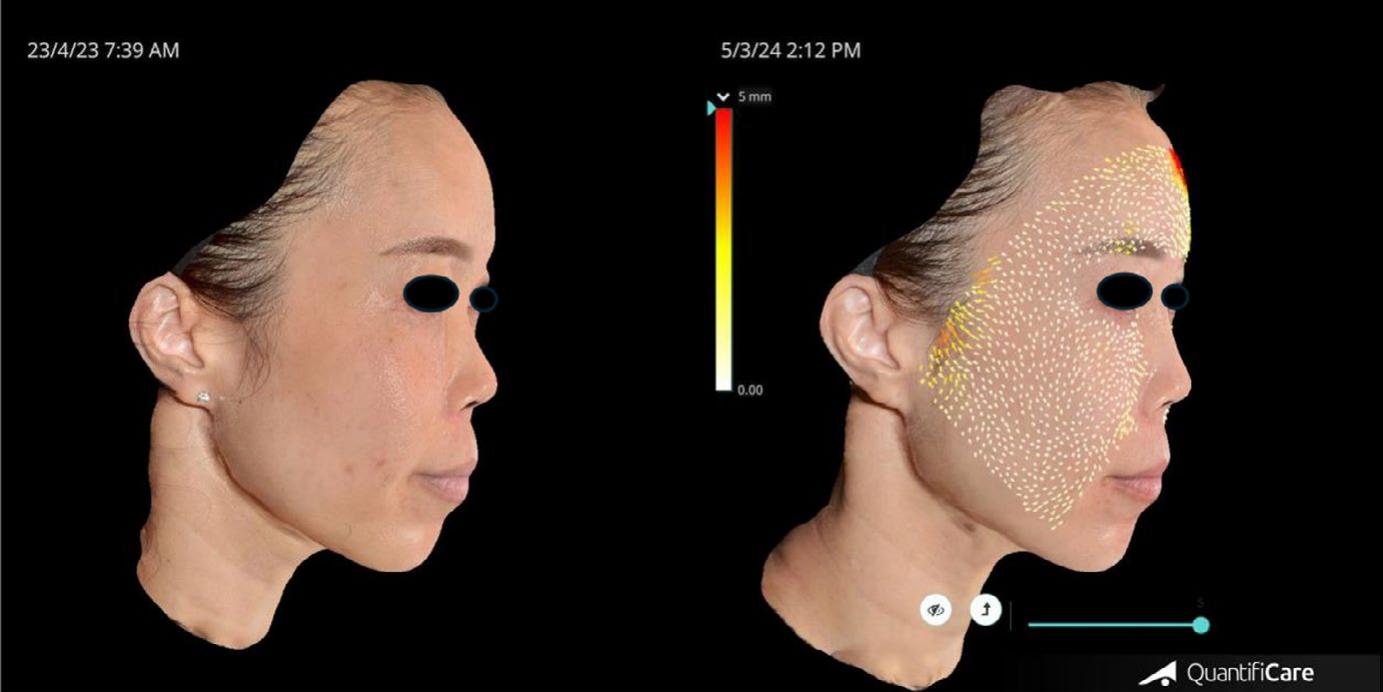
Quantificare diagram showing before and after, 11 months following treatment. Yellow arrows over the temporal area showing the temporal lift following treatment. Red arrows over the forehead demonstrating direction of forehead lift following treatment.

**FIGURE 3:**
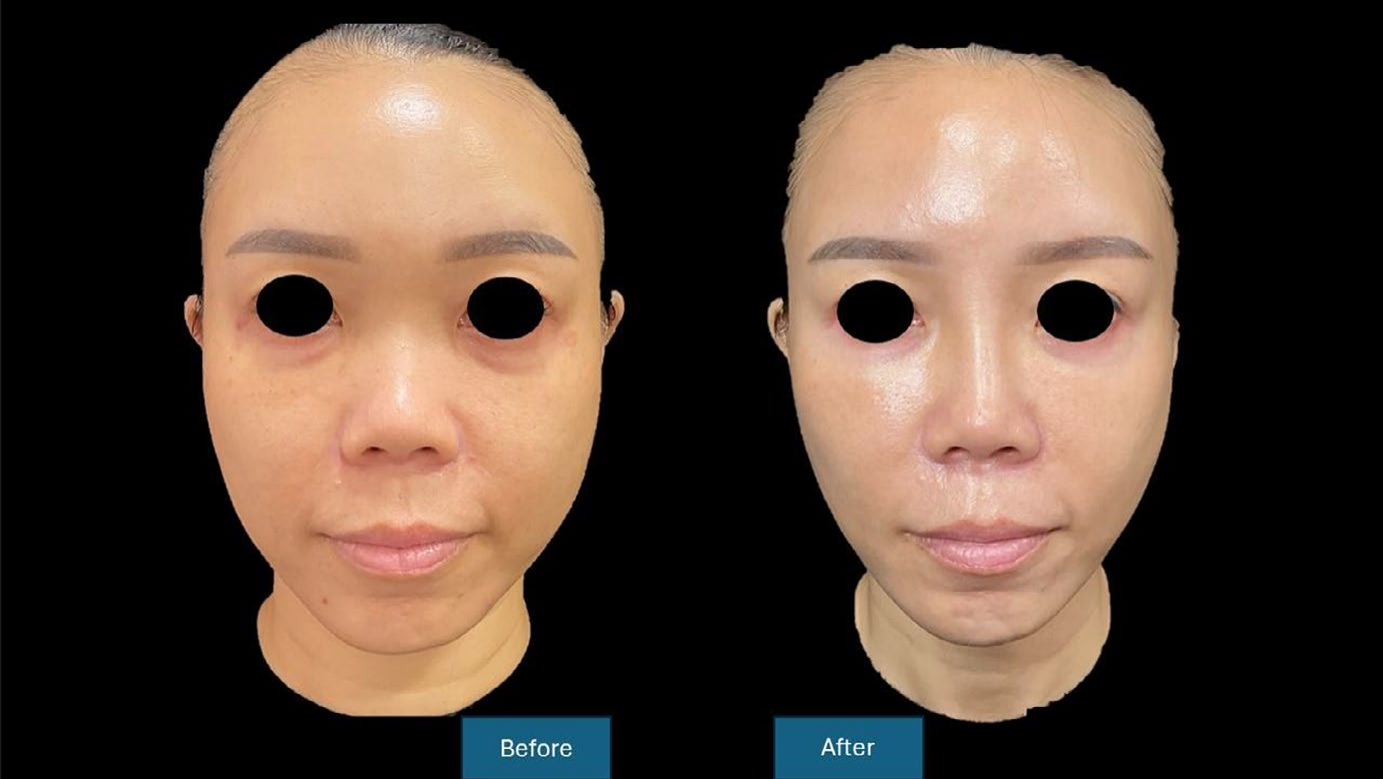
Picture showing before and after following treatment. The forehead and lower face contours have improved following treatment with combined forehead and temporal lift with Lanluma V.

**FIGURE 4:**
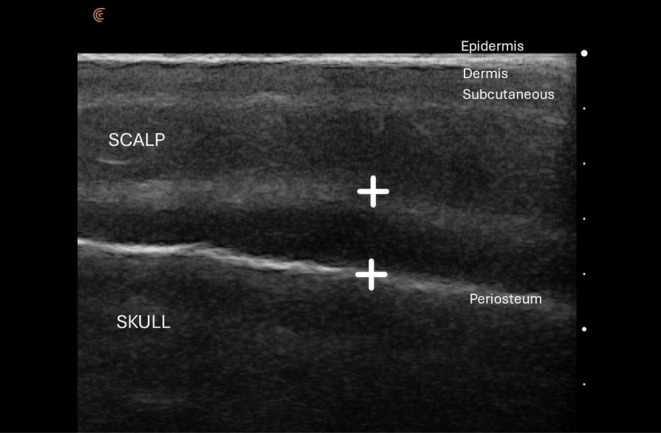
Six‐month later ultrasound picture showing layer where Lanluma V was injected over the galea.

The temporal lifting technique has been replicated and validated using hyaluronic acid [7], calcium hydroxyapatite [8] and PLLA [5]. A previously described technique, the “Crown technique” (which involves injection of 1 mL of moderate intensity hyaluronic acid on both sides of the scalp at junction of vertex and parietal aspect of scalp) and resulting in improvement of GAIS [9]. Our scalp vertex treatment involves a modification of the “Crown technique” as it involves injection of 10 mL of reconstituted Lanluma in a fan shaped pattern over the galea layer. Our technique of injecting Lanluma V using the temporal lift as well as a modification of the “Crown technique” enhances the fascial tension over the scalp and parietal region, which can be transmitted along the chain of retaining ligaments down to the jawline. This method of treatment restores the overall facial contour and rejuvenates the upper and lower parts of the face, and produces a demonstrable 1.16 average grade improvement in GAIS scores (See Figures 2, 3, 4).

The complication of non‐scarring alopecia has been identified in patients treated with hyaluronic acid and calcium hydroxyapatite over the scalp [10]. This complication, however, has not been observed in patients treated with Lanluma. The occurrence of non‐scarring alopecia has been postulated to be secondary to the highly viscous nature of hyaluronic acid [5], causing vascular occlusion; hence, a liquid collagen stimulator such as PLLA could reproduce similar results with less occurrence of such a complication.

This technique is versatile and is a valuable addition to the existing combination treatment approach toward facial rejuvenation. This combined forehead and temporal lifting technique using Lanluma V also complements other nonsurgical rejuvenation treatments such as high‐intensity focused ultrasound and radiofrequency devices to address the multiple facets of facial aging.

## 5 Summary

Combined forehead and parietal treatment with Lanluma V produces a demonstrable GAIS improvement of 1.16 on average, as assessed by a plastic surgeon. The two‐session regimen is designed to allow for gradual and sustained collagen stimulation, resulting in more natural‐looking and long‐lasting results. This innovative technique allows for combination with other nonsurgical modalities to address multiple aspects of facial aging. Lastly, there were no significant side effects such as non‐scarring alopecia detected in this series of patients.

## Conflicts of Interest

Larry Wu: none; Giovanni Salti: Consultant to Sinclair pharmaceutical.

## Data Availability

The data that support the findings of this study are available from the corresponding author upon reasonable request.
